# Validation and clinicopathologic associations of a urine-based bladder cancer biomarker signature

**DOI:** 10.1186/s13000-014-0200-1

**Published:** 2014-11-12

**Authors:** Ge Zhang, Evan Gomes-Giacoia, Yunfeng Dai, Adrienne Lawton, Makito Miyake, Hideki Furuya, Steve Goodison, Charles J Rosser

**Affiliations:** MD Anderson Cancer Center Orlando, Cancer Research Institute, Orlando, Florida USA; Department of Biostatistics, The University of Florida, Gainesville, Florida USA; Department of Pathology, Orlando Health, Orlando, FL USA; Nonagen Bioscience Corp, Orlando, Florida USA; Department of Health Sciences Research, Mayo Clinic, Jacksonville, Florida USA; University of Hawaii Cancer Center, 701 Ilalo St, Rm 327, Honolulu, HI 96813 USA

**Keywords:** Bladder cancer, Diagnosis, Grade, Signature, Stage

## Abstract

**Background:**

To validate the expression of a urine-based bladder cancer associated diagnostic signature comprised of 10 targets; ANG, CA9, MMP9, MMP10, SERPINA1, APOE, SDC1, VEGFA, SERPINE1 and IL8 in bladder tumor tissues.

**Methods:**

Immunohistochemical analyses were performed on tumor specimens from 213 bladder cancer patients (transitional cell carcinoma only) and 74 controls. Staining patterns were digitally captured and quantitated (Aperio, Vista, CA), and expression was correlated with tumor stage, tumor grade and outcome measures.

**Results:**

We revealed a positive association of 9 of the 10 proteins (excluding VEGF) in bladder cancer. Relative to control cases, a reduction in SDC1 and overexpression of MMP9, MMP10, SERPINE1, IL8, APOE, SERPINA1, ANG were associated with high stage bladder cancer. Reduced VEGF and increased SERPINA1 were associated with high-grade bladder cancer. Disease-specific survival was significantly reduced in tumors with high expression of SERPINE1 and/or IL8.

**Conclusions:**

These findings confirm that the proteins in a urine-based diagnostic signature are aberrantly expressed in bladder tumor tissues, and support the potential additional utility of selected biomarkers for the clinicopathological evaluation of excised tissue or biopsy material.

**Virtual Slides:**

The virtual slide(s) for this article can be found here: http://www.diagnosticpathology.diagnomx.eu/vs/13000_2014_200

## Background

Cancer of the urinary bladder is the fourth most common malignancy in men and the ninth most common malignancy in women in the United States [1]. Urothelial carcinomas constitute approximately 90% of all bladder cancer (BCa) cases [2]. At presentation, more than 80% of bladder tumors are non-muscle invasive bladder cancer (NMIBC, *i.e.,* Tis, Ta or T1) and the remaining 20% of bladder tumors are muscle-invasive bladder cancers (MIBC) or metastatic. NMIBC harbors a 5-year survival rate of approximately 94% [3,4], however, approximately 70% of patients with these lesions develop tumor recurrence within two years of initial diagnosis. The recurrence phenomenon of NMIBC makes it one of the most prevalent cancers worldwide (in America it is second only to colorectal cancer) and is, therefore, a great burden to healthcare systems [1,5]. Though radiation with concomitant chemotherapy is finding its place in the management of MIBC, radical cystectomy is the mainstay of treatment for these tumors, however, up to 50% of patients experience disease relapse and eventual death despite these aggressive treatment regimens. Thus, the 5-year survival rate for MIBC is approximately 50% [6,7]. These disappointing outcomes may be explained by our limited understanding of BCa tumorigenesis and progression.

The identification and validation of molecular alterations involved in BCa tumorigenesis may lead to improved diagnostic tools and improved therapeutic planning and patient management. Previously, our group has developed a novel urine-based BCa associated diagnostic signature comprised of 10 targets; angiogenin (ANG), carbonic anhydrase 9 (CA9), matrix metallopeptidase 9 (MMP9), matrix metallopeptidase 10 (MMP10), Alpha-1 Antitrypsin (SERPINA1), Apolipoprotein E (APOE), Syndecan-1 (SDC1), Vascular endothelial growth factor A (VEGFA), Plasminogen activator inhibitor-1 (SERPINE1) and Interleukin 8 (IL8) [8-12]. The utility of the signature has been confirmed in three large studies, one comprised of BCa patients and controls with diverse benign conditions, another comprised of BCa patients and controls collected from multiple sites and analyzed in an independent laboratory, and another comprised of patients on post-treatment tumor surveillance for the monitoring of recurrence [13-15]. The multiplex BCa-associated diagnostic signature has performed robustly in all of these scenarios.

In this study, we investigated the expression pattern of the urine-based signature proteins in solid bladder tumor tissue as a means to further substantiate our previously validated urine-based signature for the detection of BCa. Digital immunohistochemistry confirmed the aberrant expression of nine of the ten the biomarkers in bladder tumor tissues and revealed that selected proteins in the set had associations with stage and grade and clinical outcome.

## Methods

### Patients and clinicopathologic information

The study was performed after approval by MD Anderson Cancer Center Orlando Institutional Review Board under a request of waiver of consent on archived pathologic specimens within the Department of Pathology. The study cohort was composed of 213 patients, who underwent transurethral resection of bladder tumor, and 74 patients without a history of BCa or extensive smoking history (control), who underwent bladder biopsy for voiding dysfunction or autopsy at Orlando Health. Thus no patient had neoadjuvant chemotherapy prior to tissue collection. These paraffin embedded tissues were collected from Jan 1, 2005 to December 31, 2010. From the medical records, the following information was retrieved: age, race, sex, cancer related death. Data related to adjuvant chemotherapy was not available. Furthermore, histology (transitional cell carcinoma only), tumor grade (2002 WHO classification) and stage (2002 TNM classification) were confirmed by reevaluation of the original pathology slides. Demographic, clinical and pathologic characteristics of the 287 subjects comprising the study cohort are illustrated in Table 1.

**Table 1:** **Demographic, clinical and pathologic characteristics of the 287 subjects comprising the study cohort**

**Features**	**Bladder cancer (%) N = 213**	**Controls (%) N = 74**
**Age (years)**		
< 65	50 (23.5%)	74 (100.0%)
> 65	150 (70.4%)	0 (0.0%)
Unavailable	13 (6.1%)	
**Sex**		
Female	48 (24.0%)	16 (21.6%)
Male	152 (76.0%)	
Unavailable	13 (6.1%)	
**Race**		
Caucasian	162 (76.1%) %)	
Other	30 (14.0%)	
Unavailable	21 (9.9%)	
**Tumor grade**		
High-grade	175 (82.2%)	
Low-grade	26 (12.2%)	
Unavailable	12 (5.6%)	
**Tumor stage**		
Ta	49 (23.0%)	
Tis (CIS)	20 (9.4%)	
T1	62 (29.1%)	
T2	31 (14.6%)	
>T3, N+ or M+	39 (18.3%)	
Unavailable	12 (5.6%)	
**Recurrence**		
Yes	70 (32.9%)	
No	143 (67.1%)	

### Immunohistochemistry

Immunostaining was performed using standard protocols. Paraffin blocks were cut 5 μm sections and placed on a Superfrost Plus Microslide. Sections were deparaffinized followed by antigen retrieval using citric acid buffer (pH 6.0, 95°C for 20 min). Slides were treated with 1% hydrogen peroxide in methanol to block endogenous peroxidase activity. After 20 min blocking in 5% horse serum, slides were incubated overnight at 4°C with the following primary antibodies: anti-SERPINE1 (#HPA050039; rabbit monoclonal, dilution 1:100) from Sigma Aldrich (St. Louis, MO); anti-VEGFA (A-20) (#sc-152; rabbit polyclonal, dilution 1:500) and anti-ANG (#sc-74528; mouse monoclonal, dilution 1:10) from Santa Cruz Biotechnology, Inc. (Dallas, TX); anti-SDC1 [B-A38] (#ab34164, mouse monoclonal, dilution 1:400), anti-MMP9 [EP1254] (#ab76003, rabbit monoclonal, dilution 1:200), and anti-MMP10 (#ab38930, rabbit polyclonal, dilution 1:2000) from Abcam (Cambridge, MA); anti-CA9 (#23300002, rabbit polyclonal, dilution 1:1000) and anti-SERPINA1 (#NBP1-90309, rabbit polyclonal, dilution 1:2500) from Novus Biologicals (Littleton, CO); anti-APOE [3D12] (#M068-3, mouse monoclonal, dilution 1:200) from MBL Co. (Japan); and anti-IL8 (#AHC0881, rabbit polyclonal, dilution 1:200) from Life Technologies, Inc. (Grand Island, NY). Next, slides were incubated with 2 μg/mL of biotinylated anti-mouse or anti-rabbit IgG secondary antibody (Vector Laboratories, Burlingame, CA) for 30 min at room temperature. Subsequently, the sections were stained using Standard Ultra-Sensitive ABC Peroxidase Staining kit (Pierce/Thermo Fisher Scientific, San Jose, CA) and 3, 3′- diaminobenzidine (DAB; Vector Laboratories), counterstained by hematoxylin, dehydrated, and mounted with a cover slide.

Based on the notable reports from Human Protein Atlas (http://www.proteinatlas.org), lung (SERPINE1 and MMP9), liver (VEGF, ANG, CA9 and SERPINA1), tonsil (SDC1, MMP10 and APOE) and stomach (IL8) were used as a positive control and omitting the primary antibody served as the negative control.

### Image analysis

Immunostained slides (n = 287) were scanned into high-resolution images using the Aperio Scanscope Cs (Aperio Technologies, Vista, CA) with a 20x objective as previously described [16,17]. The images were then visualized in the software Image Scope (Aperio, Vista, CA, USA). Briefly, the location of immunoreactivity was noted. All immunostaining was cytoplasmic except SDC1, which was present in the cellular membrane or cytoplasma, based on grade or stage. All epithelial staining analyzed for this study. The selection of the regions of interest (ROI) on the training slides was initially done by an experienced pathologist (AL) and a technician (GZ) working together. After a training period, the technician, who was blinded to disease status, did the selection of the areas in cases where tumor cell areas were easily identifiable in the section. Using an algorithm developed in Aperio Scanscope Cs, staining intensity of the tissue, as well as the extent (percentage) of staining in cells was measured for each target. Then for statistical purposes, the ranked set of data for each target was divided into four groups with 1^st^ quartile having the lowest staining intensity (0 - 10%) and the 4^th^ quartile having the highest staining intensity (>50%). The quantitative immunochemistry slides were then reviewed and corroborated independently by a pathologist (AL). Whenever a discrepancy between the quantitative and semi-quantitative readings occurred, another investigator (CJR) reviewed and rendered a final score.

### Statistical analysis

SAS V9.4 (Cary, NC) was used to perform statistical analyses. The relationship between immunoexpression of the 10 targets and clinicopathological features were tested with a cross tables applying Chi-square or Fisher test, and all tests were 2-tailed. The Kaplan-Meier curves using the log-rank test were used to estimate and compare disease-specific survival. A *p* value of <0.05 was considered significant.

## Results and discussion

### Demographics of the patients and tumor characteristics

The age of the cancer patients ranged from 30 to 94 years (mean ± SD, 71.8 ± 11.9). Seventy-six percent of the cancer patients were male and 76% of the cancer patients were Caucasian. Seventy patients (33%) had a history of BCa. Twelve percent of patients had tumors larger than 5 cm, 64% had tumors between 2 and 5 cm, while 23% had tumors <2 cm (Table 1). All tumors were confirmed to be transitional cell carcinoma. The tumors were classified as either low-grade (26 [12.2%]) or high-grade (175 [82.2%]) and Tis, Ta, T1 (non-muscle invasive bladder cancer 131 [61.5%]) and T2-T4, N+, M+(muscle invasive bladder cancer 70 [32.9%]). In 12 cases, limited tissues were available for accurate stage and grade assessment.

### Immunohistochemical results

**Figure 1:**
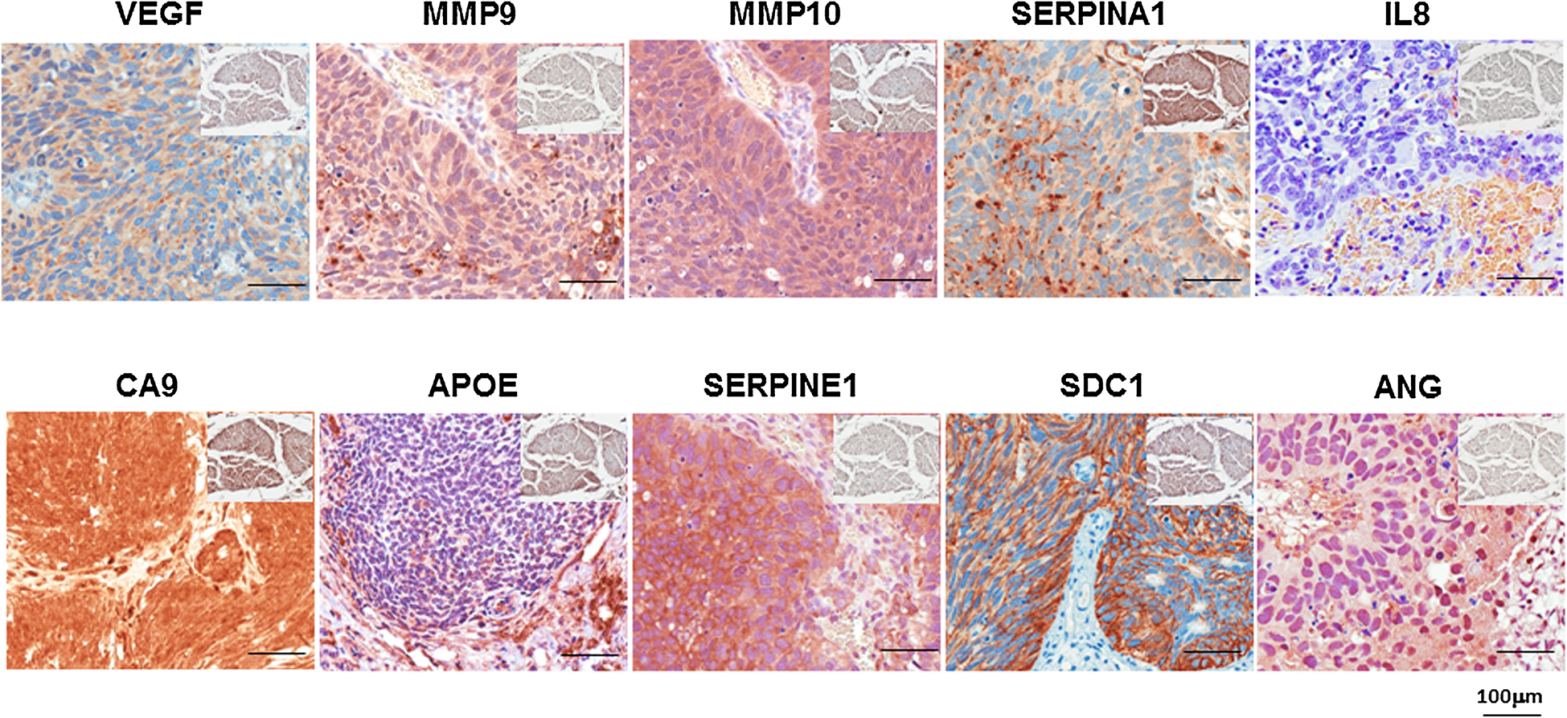
**Representative expression status for ANG, CA9, MMP9, MMP10, SERPINA1, APOE, SDC1, VEGFA, serpine1 and IL8 levels in tumor tissue.** *Insert* Representative expression status for ANG, CA9, MMP9, MMP10, SERPINA1, APOE, SDC1, VEGFA, serpine1 and IL-8 levels in benign tissue. All images were captured at 400× magnification.

Figure 1 shows representative expression status for each of the 10 targets in a high-grade non-muscle invasive tumor. The relationship between immunophenotype for each target and disease status is summarized in Table 2. Expression of 9 of the 10 biomarkers (not VEGF) showed a positive association with cancer. In our study, we found cancer cases expression levels in the 3^rd^ and 4^th^ quartile for MMP9 (66.5% *vs.* 9.5% of control), MMP10 (60.1% *vs.* 25.7% of control), SERPINE1 (57.3% *vs.* 31.9% of control), IL8 (49.5% *vs.* 41.5% of control), CA9 (57.2% *vs.* 30.0% of control), APOE (61.7% *vs.* 18.3% of control), SERPINA1 (59.6% *vs.* 25.5% of control), SDC1 (66.5% *vs.* 7.1% of control) and ANG (67.9% *vs.* 0% of control) to be significantly increased compared to control.

**Table 2:** **Relationship between immunochemical features and disease status**

**Target expression**	**Bladder cancer, %**	**Benign, %**	***p*** **-value**	**Target expression**	**Bladder cancer, %**	**Benign, %**	***p*** **-value**
**VEGFA**				**APOE**			
**1**	47 (28.7%)	12 (16.2%)	0.115	**1**	30 (15.5%)	36 (50.7%)	<0.0001
**2**				**2**	44 (22.8%)	22 (31.0%)	
**3**				**3**	54 (28.0%)	12 (16.9%)	
**4**				**4**	65 (33.7%)	1 (1.4%)	
**MMP9**				**SERPINA1**			
**1**	14 (7.7%)	50 (67.6%)	<0.0001	**1**	35 (18.8%)	30 (40.5%)	<0.0001
**2**	47 (25.8%)	17 (23.0%)		**2**	40 (21.5%)	25 (33.8%)	
**3**	60 (33.0%)	4 (5.4%)		**3**	49 (26.3%)	16 (21.6%)	
**4**	61 (33.5%)	3 (4.1%)		**4**	62 (33.3%)	3 (4.1%)	
**MMP10**				**SDC1**			
**1**	37 (20.2%)	27 (36.5%)	<0.0001	**1**	27 (14.6%)	36 (51.4%)	<0.0001
**2**	36 (19.7%)	28 (37.8%)		**2**	35 (18.9%)	29 (41.4%)	
**3**	46 (25.1%)	18 (24.3%)		**3**	60 (32.4%)	4 (5.7%)	
**4**	64 (35.0%)	1 (1.4%)		**4**	63 (34.1%)	1 (1.4%)	
**SERPINE1**				**ANG**			
**1**	47 (25.7%)	16 (22.2%)	<0.0001	**1**	4 (2.1%)	62 (86.1%)	<0.0001
**2**	31 (16.9%)	33 (45.8%)		**2**	56 (29.0%)	10 (13.9%)	
**3**	46 (25.1%)	18 (25.0%)		**3**	66 (34.2%)	0 (0%)	
**4**	59 (32.2%)	5 (6.9%)		**4**	67 (34.7%)	0 (0%)	
**IL8**							
**1**	56 (28.6%)	10 (24.3%)	<0.0001				
**2**	43 (21.9%)	24 (34.3%)					
**3**	36 (18.4%)	30 (32.9%)					
**4**	61 (31.1%)	6 (8.6%)					
**CA9**							
**1**	35 (17.9%)	31 (44.3%)	<0.0001				
**2**	49 (25.0%)	18 (25.7%)					
**3**	48 (24.5%)	18 (25.7%)					
**4**	64 (32.7%)	3 (4.3%)					

The relationship between immunophenotype for each target and tumor grade is summarized in Table 3. We found only high-grade disease expression level in the 3^rd^ and 4^th^ quartile for SERPINA1 (62.4% *vs.* 45.4% of low-grade) to be significantly increased compared to low-grade disease. Interestingly, we found that VEGFA expression levels in the high-grade disease 3^rd^ and 4^th^ quartile were significantly reduced compared to low-grade disease (40.3% *vs.* 70.9, respectively, *p* =0.031). The relationship between immunophenotype for each target and tumor stage is summarized in Table 4. High stage disease correlated with increased expression level (*i.e.,* more 3^rd^ and 4^th^ quartile immunostaining) for MMP9, MMP10, SERPINE1, IL8, APOE, SERPINA1 and ANG. As we have previously reported [18], a shift in cellular location of SDC1 (membrane to cytoplasm) was noted in high stage disease. This change in the location of SDC1 protein, resulted in an inverse association with SDC1 cell membrane expression level.

**Table 3:** **Relationship between immunochemical features and tumor grade**

**Target expression**	**Low-grade, %**	**High-grade, %**	***p*** **-value**	**Target expression**	**Low-grade, %**	**High-grade, %**	***p*** **-value**
**VEGFA**				**APOE**			
**1**	4 (16.7%)	43 (30.9%)	0.031	**1**	6 (25.0%)	24 (14.7%)	0.598
**2**	3 (12.5%)	40 (28.8%)		**2**	5 (20.8%)	39 (23.9%)	
**3**	7 (29.2%)	30 (21.6%)		**3**	5 (20.8%)	46 (28.2%)	
**4**	10 (41.7%)	26 (18.7%)		**4**	8 (33.3%)	54 (33.1%)	
**MMP9**				**SERPINA1**			
**1**	3 (12.5%)	10 (6.5%)	0.161	**1**	2 (9.1%)	32 (20.4%)	0.021
**2**	9 (37.5%)	36 (23.5%)		**2**	10 (45.5%)	27 (17.2%)	
**3**	8 (33.3%)	50 (32.7%)		**3**	5 (22.7%)	43 (27.4%)	
**4**	4 (16.7%)	57 (37.3%)		**4**	5 (22.7%)	55 (35.0%)	
**MMP10**				**SDC1**			
**1**	7 (29.2%)	29 (19.0%)	0.611	**1**	2 (8.0%)	25 (15.7%)	0.222
**2**	3 (12.5%)	32 (20.9%)		**2**	3 (12.0%)	31 (19.5%)	
**3**	6 (25.0%)	39 (25.5%)		**3**	7 (28.0%)	53 (33.3%)	
**4**	8 (33.3%)	53 (34.6%)		**4**	13 (52.0%)	50 (31.4%)	
**SERPINE1**				**ANG**			
**1**	10 (41.7%)	34 (22.4%)	0.213	**1**	0 (0%)	4 (2.5%)	0.783
**2**	4 (16.7%)	27 (17.8%)		**2**	7 (29.2%)	47 (28.8%)	
**3**	5 (20.8%)	38 (25.0%)		**3**	7 (29.2%)	56 (34.4%)	
**4**	5 (20.8%)	53 (34.9%)		**4**	10 (41.7%)	56 (34.4%)	
**IL8**							
**1**	9 (37.5%)	43 (26.2%)	0.699				
**2**	4 (16.7%)	37 (22.6%)					
**3**	4 (16.7%)	32 (19.5%)					
**4**	7 (29.2%)	52 (31.7%)					
**CA9**							
**1**	7 (30.4%)	28 (16.9%)	0.206				
**2**	6 (26.1%)	40 (24.1%)					
**3**	2 (8.7%)	43 (25.9%)					
**4**	8 (34.8%)	55 (33.1%)

**Table 4:** **Relationship between immunochemical features and tumor stage**

**Target expression**	**Ta**	**Tis (CIS)**	**T1**	**T2**	**>T3, N+ or M+**	***p*** **-value**	**Target expression**	**Ta**	**Tis (CIS)**	**T1**	**T2**	**>T3, N+ or M+**	***p*** **-value**
**VEGFA**							**APOE**						
**1**	10 (25.0%)	5 (29.4%)	18 (33.3%)	6 (25.0%)	8 (28.6%)	0.670	**1**	13 (27.7%)	1 (6.3%)	9 (15.3%)	7 (24.1%)	0 (0%)	0.009
**2**	6 (15.0%)	5 (29.4%)	17 (31.5%)	6 (25.0%)	9 (32.1%)		**2**	14 (29.8%)	6 (37.5%)	14 (23.7%)	5 (17.2%)	5 (13.9%)	
**3**	10 (25.0%)	4 (23.5%)	9 (16.7%)	7 (29.2%)	7 (25.0%)		**3**	10 (21.3%)	5 (31.3%)	13 (22.0%)	6 (20.7%)	17 (47.2%)	
**4**	14 (35.0%)	3 (17.6%)	10 (18.5%)	5 (20.8%)	4 (14.3%)		**4**	10 (21.3%)	4 (25.0%)	23 (39.0%)	11 (37.9%)	14 (38.9%)	
**MMP9**							**SERPINA1**						
**1**	5 (11.6%)		4 (7.1%)	4 (13.8%)		0.036	**1**	11 (25.0%)	2 (13.3%)	16 (27.6%)	3 (10.7%)	2 (5.9%)	0.036
**2**	15 (34.9%)	5 (35.7%)	16 (28.6%)	4 (13.8%)	5 (14.3%)		**2**	10 (22.7%)	2 (13.3%)	12 (20.7%)	9 (32.1%)	4 (11.8%)	
**3**	13 (30.2%)	5 (35.7%)	22 (39.3%)	7 (24.1%)	11 (31.4%)		**3**	10 (22.7%)	4 (26.7%)	19 (32.8%)	6 (21.4%)	9 (26.5%)	
**4**	10 (23.3%)	4 (28.6%)	14 (25.0%)	14 (48.3%)	19 (54.3%)		**4**	13 (29.5%)	7 (46.7%)	11 (19.0%)	10 (35.7%)	19 (55.9%)	
**MMP10**							**SDC1**						
**1**	11 (25.6%)	8 (57.1%)	12 (21.8%)	2 (6.9%)	3 (8.3%)	0.036	**1**	6 (14.3%)	4 (21.1%)	5 (8.5%)	5 (17.2%)	7 (20.0%)	0.021
**2**	9 (20.9%)	2 (14.3%)	10 (18.2%)	8 (27.6%)	6 (16.7%)		**2**	9 (21.4%)	6 (31.6%)	5 (8.5%)	8 (27.6%)	6 (17.1%)	
**3**	10 (23.3%)	3 (21.4%)	12 (21.8%)	9 (31.0%)	11 (30.6%)		**3**	13 (31.0%)	6 (31.6%)	17 (28.8%)	8 (27.6%)	16 (45.7%)	
**4**	13 (30.2%)	1 (7.1%)	21 (38.2%)	10 (34.5%)	16 (44.4%)		**4**	14 (33.3%)	3 (15.8%)	32 (54.2%)	8 (27.6%)	6 (17.1%)	
**SERPINE1**							**ANG**						
**1**	19 (45.2%)	8 (53.3%)	12 (21.4%)	2 (7.1%)	3 (8.6%)	0.0003	**1**	0 (0%)	1 (5.9%)	0 (0%)	3 (10.3%)	0 (0%)	<0.0001
**2**	7 (16.7%)	1 (6.7%)	11 (19.6%)	4 (14.3%)	8 (22.9%)		**2**	23 (50.0%)	8 (47.1%)	16 (27.1%)	6 (20.7%)	1 (2.8%)	
**3**	9 (21.4%)	5 (33.3%)	15 (26.8%)	5 (17.9%)	9 (25.7%)		**3**	12 (26.1%)	2 (11.8%)	24 (40.7%)	10 (34.5%)	15 (41.7%)	
**4**	7 (16.7%)	1 (6.7%)	18 (32.1%)	17 (60.7%)	15 (42.9%)		**4**	11 (23.9%)	6 (35.3%)	19 (32.2%)	10 (34.5%)	20 (55.6%)	
**IL8**													
**1**	19 (41.3%)	7 (43.8%)	20 (33.3%)	4 (13.8%)	2 (5.4%)	0.001							
**2**	12 (26.1%)	3 (18.8%)	16 (26.7%)	4 (13.8%)	6 (16.2%)								
**3**	6 (13.0%)	1 (6.3%)	11 (18.3%)	10 (34.5%)	8 (21.6%)								
**4**	9 (19.6%)	5 (31.3%)	13 (21.7%)	11 (37.9%)	21 (56.8%)								
**CA9**													
**1**	12 (26.7%)	4 (23.5%)	10 (16.7%)	7 (24.1%)	2 (5.3%)	0.308							
**2**	12 (26.7%)	5 (29.4%)	9 (15.0%)	6 (20.7%)	14 (36.8%)								
**3**	9 (20.0%)	3 (17.6%)	17 (28.3%)	6 (20.7%)	10 (26.3%)								
**4**	12 (26.7%)	5 (29.4%)	24 (40.0%)	10 (34.5%)	12 (31.6%)								

### Immunophenotype and survival

**Figure 2:**
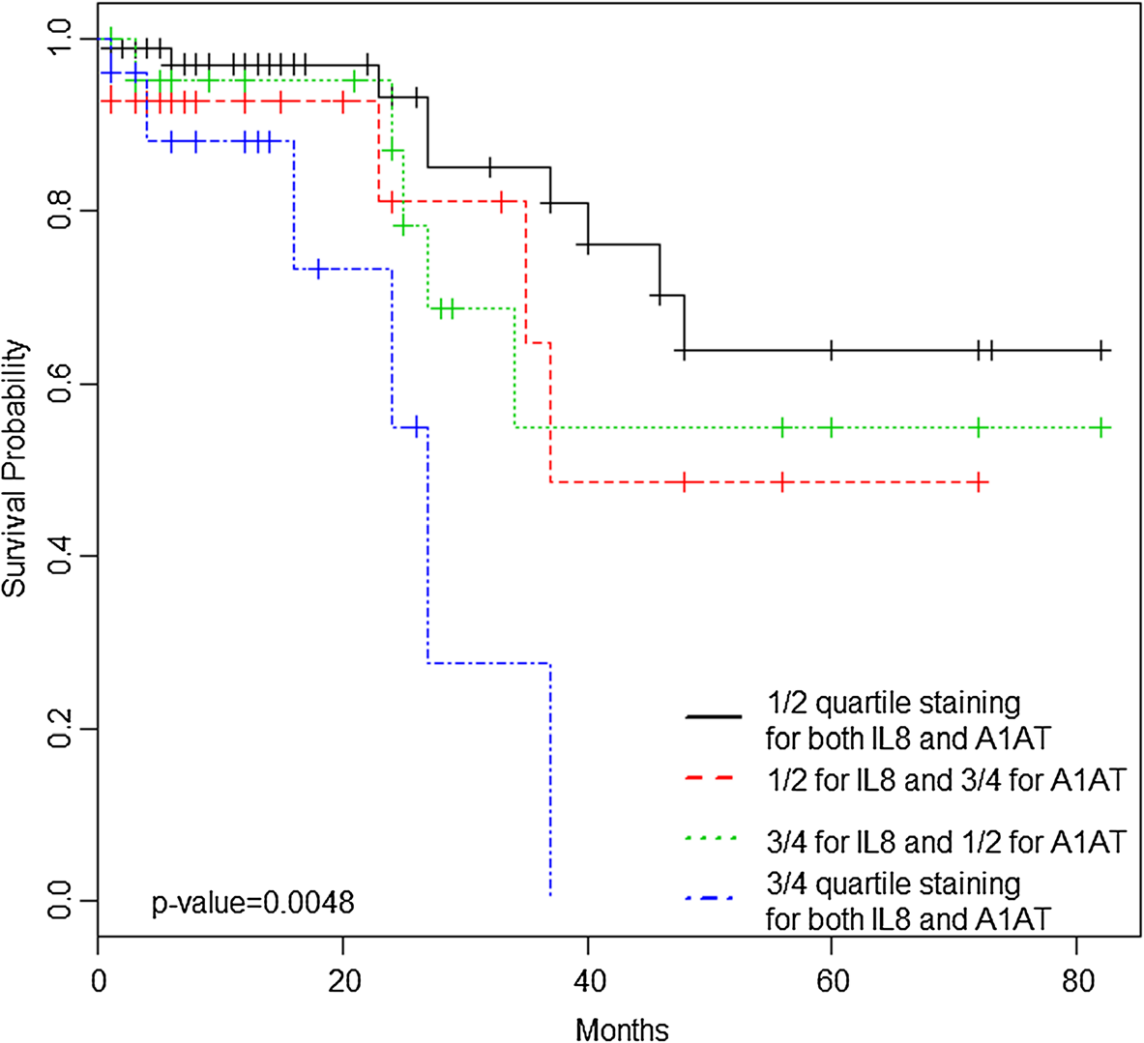
**Univariate analysis of the prognostic impact of SERPINA1 and IL8 co-over-expression on disease-specific survival of bladder cancer patients.**

The follow-up period for the cohort ranged from 1 to 82 months (median 6 months), and the mean survival time was 16 months. Using Kaplan-Meier survival analysis with the log-rank test, we found significantly worse disease-specific survival (DSS) when the expression levels of SERPINA1 or IL8 were increased. On multivariate analysis when controlling for stage and grade, biomarker levels did not independently predict DSS (data not shown). For statistical purposes when analyzing KM curves for the combination of SERPINA1 and IL8, quartile 1 and 2 were combined and compared to the combination of quartile 3 and 4. Based on these results, we re-analyzed SERPINA1 and IL8 immunostaining in the following combinations: SERPINA1 1^st^/2^nd^ quartile with IL8 1^st^/2^nd^ quartile; SERPINA1 3^rd^/4^th^ quartile with IL8 1^st^/2^nd^; SERPINA1 1^st^/2^nd^ quartile with IL8 3^rd^/4^th^ quartile; SERPINA1 3^rd^/4^th^ quartile with IL8 3^rd^/4^th^ quartile. When both SERPINA1 and IL8 expression were increased, survival was significantly reduced compared to assessment of SERPINA1 alone or IL8 alone (*p* =0.0048) (Figure 2).

Previously, we have employed proteomic [19,20] and genomic analyses [21,22] of urine components to identify accurate and robust molecular signatures for the non-invasive diagnosis of BCa. A candidate 14-biomarker urinary protein signature was subsequently tested in an independent cohort composed of 127 patients (64 BCa) [8-12]. That study narrowed the diagnostic signature to 10 biomarkers (IL8, MMP9, MMP10, SERPINA1, VEGFA, ANG, CA9, APOE, SDC1 and SERPINE1), and this was validated in a cohort of 308 patients (102 BCa) with varying benign urologic diagnoses [13]. Furthermore, using an independent test laboratory, we externally validated these results in a multi-institute cohort comprised of 320 patients (183 BCa). The 10-biomarker signature achieved 79%, specificity of 79% (AUROC 0.846) for non-invasive BCa detection in phase II external validation study [14]. Based on these results, the multiplex urine-based signature is the focus of development into a clinical test.

In our previous work, the non-invasively obtained material (urine) was directly subjected to molecular profiling for biomarker discovery. This strategy was chosen to avoid the potential drop-out of biomarkers that can occur when translating from tissue-based discovery studies [23-25] to biological fluids for assay development. A number of tissue-based biomarkers have translated to urinalysis [26-28], but translation can be affected by secretion rate, enzymatic breakdown or the stability of the protein in the dilute media. Furthermore, urine as a discovery material also has the advantages of being relatively easy to obtain, including the chance for serial sampling, and because of its relatively low complexity compared to solid tissue or blood.

Here, we investigated the expression patterns of our urine-based biomarker panel in excised bladder tumor tissue. Of the 10 biomarkers tested, all but VEGFA had overexpression of the biomarkers compared to control tissues. Additionally, elevated levels of SERPINA1 and reduced levels of VEGFA were associated with high-grade BCa, and elevated levels of MMP9, MMP10, SERINE1, IL8, APOE, SERPINA1 and ANG (and reduced levels of SDC1) were associated with high stage BCa. High expression levels of SERPINA1 and IL8 were associated with a reduction in disease-specific survival, and when both SERPINA1 and IL8 were highly expressed there was a significant reduction in survival (*p = 0.0048).* Furthermore, many of these biomarkers are cytokines (*e.g.* IL8 and VEGF) thus ‘bleeding’ of biomarkers into stroma would be expected and was noted. These findings validate the inclusion of the majority of the urine-based biomarkers in a non-invasive BCa diagnostic signature but also suggest that some of the same biomarkers may have utility in prognostic evaluation when monitored in solid tissue and perhaps in urine.

A number of molecular changes have been associated with development and progression of BCa. Such molecular changes include 1) alterations in expression and regulation of the receptor tyrosine kinases, fibroblast growth factor receptor 3, and members of the epidermal growth factor receptor family, 2) upregulation of signaling through RAS and phosphatidylinositol 3-kinase/AKT pathways, and 3) functional down-regulation of the tumor suppressors, p53, pRb, and p16 through deletion, mutation and/or silencing [29-31]. Thus further understanding the molecular mechanisms underpinning the development of aggressive tumor growth in BCa is pivotal to 1) understand tumor biology, 2) effectively diagnosing BCa, 3) exploiting the prognostic capabilities and 4) identifying novel targets for pharmacological intervention.

The biomarkers that compose the diagnostic signature have a varied range of ascribed functions including angiogenesis, breakdown of extracellular matrix, serine protein inhibitor, catalyze the reversible hydration of carbon dioxide, lipoprotein metabolism and cell binding/signaling (Table 5) with the two principal ascribed functions, angiogenesis (IL8, VEGFA and ANG) and breakdown of extracellular matrix (MMP9 and MMP10). In fact, MMP9, MMP10 and SERPINE1 [32-34] have also been associated with angiogenesis. Angiogenesis, the development of new blood vessels from existing blood vessels, is essential for normal growth and development of tissues and organs. A balance of pro-angiogenic factors and anti-angiogenic factors tightly controls this process [35-37]. However in solid tumors, the balance may favor pro-angiogenic factors and thus the ability to sustain this abnormal growth of tissue [38]. Furthermore in addition to MMP9 and MMP10 degrading extracellular matrix, recent studies have suggested that ANG and SERPINE1 can breakdown the extracellular matrix [39,40]. Degradation of the extracellular matrix allows cells to become more motile. Thus, working in conjunction with the increase in vasculature to increase the probability that motile-invasive tumor cells may enter the circulation to disseminate to distant organs [41]. The extent of tumor vascularization differs between malignancies, and has been shown to correlate directly with metastatic potential [42].

**Table 5:** **Annotated urine-based bladder cancer associated diagnostic**

**Full name**	**Abbreviation**	**Ascribed function**	**Location**	**Interacts with other members of signature**
Interleukin 8	IL8	chemoattractant & angiogenesis	Extracellular	MMP9, SDC1
Angiogenin	ANG	angiogenesis	Extracellular, nucleus	None
Vascular endothelial growth factor A	VEGFA	angiogenesis	Extracellular, cytoplasm	None
Matrix metallopeptidase 9	MMP9	breakdown of extracellular matrix	Extracellular	IL8, MMP10
Matrix metallopeptidase 10	MMP10	breakdown of extracellular matrix	Extracellular	MMP9
Serpin peptidase inhibitor	SERPINA1	serine protease inhibitor	Extracellular	None
Serpin peptidase inhibitor	SERPINE1	serine endopeptidase inhibitor	Extracellular, plasma membrane	None
Carbonic anhydrase IX	CA9	catalyze the reversible hydration of carbon dioxide	Plasma membrane	None
Apolipoprotein E	APOE	Lipoprotein catabolism and metabolism	Extracellular, plasma membrane, cytoplasm	None
Syndecan 1	SDC1	cell binding, cell signaling, cytoskeletal organization	Plasma membrane, cytoplasm	IL8

Our study has important limitations. First, although this is a rather large study with specimens from 213 BCa patients being analyzed, when assessing 10 targets we would ideally like to have an even larger cohort. Second, the majority of our tumors assessed were high-grade tumors, which reflects the tertiary nature of our facility. Next due to the limited dataset, we could not evaluate these biomarkers for distinguishing progressive NMIBC from non-progressive NMIBC as well as progressive MIBC from non-progressive MIBC. With this study serving as proof of principle, we are now in the process of designing a larger, prospective study that can assess these factors. Control cohort is not age matched to BCa cohort. This is not surprising since the average age for bladder cancer patient undergoing transurethral bladder resection was 71 years while the average age for controls undergoing bladder biopsy for voiding dysfunction was 39 years. We believe this age discrepancy is of limited clinical significance. Ideally, we would have had corresponding urine to analyze the biomarker signature via ELISA to document elevated urinary as well as tissue biomarkers in this cohort. However we do not think this distracts since we have previously validated the urinary levels of the signature in over 800 clinical samples [12-15] studies to directly compare urinary protein levels with immunoreactivity levels.

Clinically, accurate BCa assays may have a clear impact on initial diagnostic performance, and on the long-term clinical management of BCa patients. If reliable urinary diagnostic biomarker assays can reduce the number of invasive and uncomfortable cystoscopies, then improvements in patient compliance and satisfaction will likely follow. Furthermore, the increased diagnostic efficiency and cost-savings from such assays will benefit both the patients and the healthcare systems. The ultimate goal is to be able to detect BCa in a timely manner such that the patient can expect an improved survival as well as improved quality of life.

## Conclusions

BCa disease management is hampered by lack of diagnostic or prognostic markers capable of a) predicting disease and b) predicting the likely disease course. Thus, there is an urgent need for identification and characterization of the molecular alterations that underlie lethal disease and that identifies more aggressive tumors early for radical and/or novel therapies. The present work shows that the expression patterns of the biomarkers in our BCa-associated diagnostic signature are largely reflected in solid tumor tissues and specific associations with grade and stage and DSS were revealed. A combination of these immunohistochemical biomarkers may aid in tissue evaluation with respect to diagnosis of malignancy or in predicting the behavior of individual BCa cases, and if confirmed, some of the targets may represent potential therapeutic targets for human urothelial cancer.

## Abbreviations

ANG: Angiogenin
CA9: Carbonic anhydrase 9
MMP9: Matrix metallopeptidase 9
MMP10: Matrix metallopeptidase 10
SERPINA1: Alpha-1 Antitrypsin
APOE: Apolipoprotein E
SDC1: Syndecan-1
VEGFA: Vascular endothelial growth factor A
SERPINE1: Plasminogen activator inhibitor-1
IL8: Interleukin 8
BCa: Bladder cancer
NMIBC: Non-muscle invasive bladder cancer
MIBC: Muscle invasive bladder cancer
DSS: Disease specific survival
AUROC: Area under receiver operator curve
